# Characteristics of orthognathic multidisciplinary team clinics in England. Part 2: A qualitative study

**DOI:** 10.1177/14653125231165069

**Published:** 2023-03-30

**Authors:** Ninu R Paul, Sarah L Rolland

**Affiliations:** Department of Orthodontics, School of Dental Sciences, Newcastle University, Newcastle upon Tyne, UK

**Keywords:** clinician’s perspective, consultant orthodontist, multidisciplinary teams, orthognathic

## Abstract

**Objective:**

The aim of the study was to explore the orthodontist’s perspective of the strengths and weaknesses of their current multidisciplinary team (MDT) design for orthognathic treatment.

**Participants and methods:**

This was a qualitative study using online interviews of orthodontic consultants across England. The data were analysed using thematic analysis. This was the second part of a two-part study, where the first part, an online questionnaire survey, identified the variation in design of orthognathic MDTs across England and was the source of the 19 participants for this study.

**Results:**

Seven themes were identified that influenced the design of orthognathic MDTs across England. Close working relationship with the team, access to closed surgery space to conduct the MDT and access to 3D planning were identified as definite strengths of some MDT clinics. Lack of a team psychologist and long waiting lists were identified as weaknesses of some orthognathic MDTs. Teaching and training within MDT clinics were highlighted as a strength of MDTs where surgery spaces were not restricted due to the pandemic. Finally, there was general agreement about revising the orthognathic minimum dataset for records collection as it was not thought to be in the patient’s best interest.

**Conclusion:**

This study was able to identify key areas considered to be determinants of a successful orthognathic MDT design from the orthodontic consultant’s perspective. Orthodontic consultants across England prioritised the need for a psychologist in the orthognathic MDT to improve the effectiveness of these clinics.

## Introduction

Orthognathic treatment is recommended to be provided through a multidisciplinary team (MDT) clinic (Royal College of Surgeons of England, 2013). The gold standard for an orthognathic MDT should include an orthodontist, a maxillofacial surgeon and a psychologist to provide holistic care (Casey et al., 2021; Cunningham and Johal, 2015). However, many orthognathic units do not have a psychologist as part of the orthognathic MDT (Paul, 2017). A marked variation in the orthognathic care pathway has been reported (Morris, 2006; Parbatani et al., 2010) and confirmed via a questionnaire study preceding this study by the same team. Further, Ireland et al. (2019) analysed the acceptance criteria, information provision and attendance of MDT clinics by orthognathic patients in an audit. This audit reported that 3.07% of patients had not been through the pre-treatment MDT. This finding shows a lack of strict adherence to a preoperative orthognathic MDT clinic attendance for all patients.

In other medical specialties, such as oncology and cleft lip and palate services, there is evidence of well-organised structures for MDT services (Clinical Standards Advisory Group [CSAG], 1998). One study of a head and neck cancer MDT used a qualitative method to identify barriers in effective patient involvement in decision-making (Hamilton et al., 2016) indicating qualitative methods could be useful in evaluating MDT design and therefore identifying facilitators and barriers in the orthognathic process of patient care.

Interestingly, no study to date has assessed the effectiveness of the design of a multidisciplinary orthognathic clinic, and the clinician’s perspective about orthognathic clinics has not been explored. This information would enable the identification of facilitators and barriers in orthognathic care pathways, improving the standard of patient care and quality of services.

## Aim

The aim of the present study was to explore the clinician’s perspective of the strengths and weaknesses of their current MDT design for orthognathic treatment. The objectives were: (1) identify the features of orthognathic MDT that are valued by orthodontists; and (2) identify negatively influencing features of an orthognathic MDT.

## Participants and methods

Ethical approval for this study was obtained from Newcastle University Ethics Committee (Ref No. 4561/2020).

This was a qualitative study using online semi-structured interviews and the second part of a two-part mixed-methods study. The first part was an online questionnaire study that aimed to identify the variance in the orthognathic MDT design used across England. This was conducted by inviting orthodontic consultants involved in orthognathic MDTs to respond to the questionnaire via the British Orthodontic Society – Consultant Orthodontist Group mailing list. The questionnaire ended with asking respondents to indicate a willingness to be contacted for this second part of the study by providing their contact email address. Thus, all 27 orthodontic consultants who expressed willingness to be interviewed were contacted to participate in an online interview of 30–60 min. Convenience sampling was undertaken to include all the orthodontic consultants involved in orthognathic MDTs who were available and willing for the interview, so as to gather a deeper understanding of subjective and location-specific information from across England. All 27 consultants were contacted on two separate occasions via email requesting availability for interview. Only one consultant responded with a refusal to participate due to lack of time. A total of 20 consultants responded with availability for online interviews and signed consent forms. The one-to-one online (Zoom/MS Teams) interviews were recorded and transcribed verbatim.

All interviews were conducted by one researcher (NP) who has training in and experience of using in-depth interviews and qualitative data analysis. The interviewer was introduced to the participant as a researcher with interest in orthognathic patient care pathway. A topic guide was used to organise the structure of the interviews, but participants were free to talk about any other relevant aspects. Simultaneous to data collection, an analysis of the data was carried out, and any new themes identified were explored further in subsequent interviews.

Data were analysed using thematic analysis using the framework approach. This was done by following six steps (Braun and Clarke, 2006). First, the researcher (NP) familiarised herself with the data by listening to the audio recordings of the interviews. The transcripts were coded, and later themes were identified from the table listing all the codes. These themes were reviewed and defined in consultation with the research team. Once all the themes and sub-themes were identified, findings were written up.

The primary researcher who conducted the interviews and analysed data was not directly part of an orthognathic team. This enabled the researcher to be objective, take an outsider’s view on the orthognathic MDTs and reduce bias. Other members on the research team with the day-to-day experience of orthognathic MDT enabled fact-checking of the data collected and analysed. This improved the reflexivity and robustness of the study.

## Results

**Figure 1.**
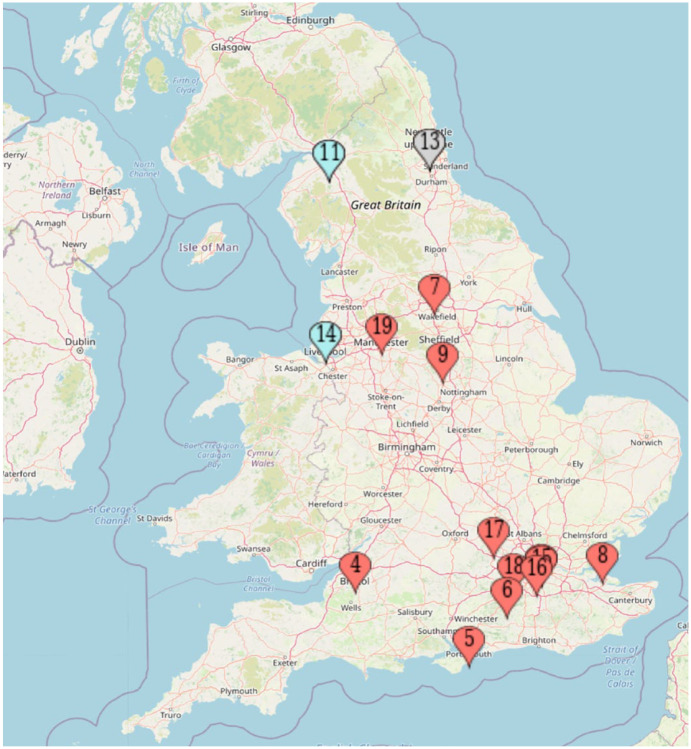
Map showing the location of the participants in the sample.

Out of the 20 interviews conducted, one was excluded since the consultant had not participated in any recent orthognathic MDT clinics. Therefore, data obtained from the interviews of 19 orthodontic consultants (10 men, 9 women) involved in orthognathic patient care from various parts of England were included in the data analysis. Figure 1 shows the spread of the orthodontic consultants on the map who were interviewed for this study. Table 1 details the demographics of the participants. Although the 16th interview attained saturation of the data, further data collection via online interviews was carried out to obtain a wealth of data about individual orthodontists’ perspectives of the orthognathic MDT design. This will be used in subsequent qualitative research by the primary researcher.

**Table 1.** Participant demographics.

Location	Participant demographics
Buckinghamshire	1 woman
Cumbria	1 man, 2 women
Derbyshire	1 man
Essex	1 man
Greater Manchester	1 woman
Hampshire	1 woman
London	1 woman, 2 men
Merseyside	1 man, 1 woman
Somerset and Gloucestershire	1 man
Tyne and Wear	1 man, 1 woman (1 man excluded)
West Yorkshire	1 man

The findings of this qualitative part of the study confirmed the variation in the orthognathic MDT design across England, already identified in the first quantitative part. The variation was not only limited to the structure of the MDT clinics but also dependent on the various type of funding available for the orthognathic service across England.

‘*Now the Commissioners commission orthognathic surgery different in the different regions [. . .] We’ve had patients, where they didn’t qualify for treatment here and then went somewhere else and got treatment and it causes real difficulties and it’s very, very difficult*.’ (P3, man)

Seven themes were identified when exploring the strengths and weaknesses of the various orthognathic MDT.

### Theme 1: Close working relationship with the team

A close working relationship based on trust and familiarity of the orthodontist with the surgeon was found to be a strength of the orthognathic MDT. This was positively reported by 13 participants. The majority of the participants did not have a clinical psychologist in the team, but the orthodontist valued the psychological input and appreciated their teamwork when one was present.

‘*Something that I like about the clinic is that we see the pre-op patients jointly, and we do the surgical plan with the orthodontist is usually like worked up an idea of what they think the plan might be. And then that will be appraised by Mr [Surgeon] and amended as needed, and so we do that, on the joint clinic as well*.’ (P4, man)

Orthodontists valued the surgeon’s outcome predictability based on their familiarity working with the surgeon.

‘*I have worked with Mr [Surgeon] for many years and for me his outcome is very predictable. This makes it very easy for me to work with him.*’ (P13, woman)

‘*I’ve been working with my max fax surgeon for quite some time, so we have a sort of symbiotic relationship with each other, so I generally introduce myself and the max fax surgeon to the patient in the joint clinic*.’ (P10, man)

This familiarity the orthodontist had with the surgeons reflects on the running of the joint clinic.

### Theme 2: Access to closed surgery space

The setting in which the orthognathic MDT was conducted was considered an important aspect of the clinic’s success. The majority of the participants reported satisfaction with the clinical setting available to them. Only two participants reported using open plan clinics for orthognathic MDT and were happy with this setting. In general, orthodontists favoured a sufficiently big enough closed surgery space for successfully conducting the orthognathic MDT clinics. The reasons identified for this were to allow privacy for the patients to discuss their concerns with the clinical team and for clinicians to have enough space available without overcrowding.

‘*[Open clinics] – it presents its challenges. And increasingly with our clinical psychologist to kind of quite difficult to have read into the personal conversations, especially if the screening questionnaires have raised challenges. We have ways to deal with those however; we have access to enclosed rooms as a single side surgery or other offices in the area we need to have those sort of one-to-one private discussions*.’ (P9, man)

However, considering the pandemic, with social distancing, orthodontists have expressed concerns about having access to a room big enough to accommodate trainees in the MDT clinics to improve teaching and training.

‘*It’s fine, really, yeah yeah, it’s just a bit smaller than we would need to get a registrar in because of social distancing, but you know, in normal circumstances, it’s a decent size, yeah. . . So, it’s a pretty good-sized dental surgery really*.’ (P15, man)

This leads to the next theme, which can be a strength or weakness of the MDT design based on the setting and personnel involved in the MDT.

### Theme 3: Teaching and training opportunity associated with an orthognathic MDT

Orthognathic MDT clinics were identified as a great avenue for teaching and training senior orthodontic trainees by 11 participants. Consultants who could accommodate orthodontic trainees in the MDT clinics expressed satisfaction, and those who had limitations, such as availability of space in the surgery, expressed dissatisfaction.

‘*They’re [MDTs] fantastic for the orthodontic trainees but they’re not so good for the surgical trainees, because they never seem to want to get involved.*’ (P16, man)

‘*I think, with regards to that part of teaching and training, what we do, could be better, to be honest it’s not it’s not ideal*.’ (P17, man)

Yet another aspect identified to affect the teaching and training on MDT clinics was the smooth running of clinics without being late

‘*If clinic always run late, it was a missed teaching opportunity for trainees. . .*’ (P7, woman)

### Theme 4: Waiting lists for treatment and surgery

In total, 10 participants reported the length of the waiting list as a cause of concern both to commence orthognathic treatment and to have surgery.

‘*So it [waiting time] wasn’t two years, but it was over a year, so you know again that was too long, I would say that we really want to be getting operations for people in three to six months, I mean I don’t I don’t think it should be any longer than that really [. . .] with a lot of cancel lists and you know, then we started to struggle with the you know the time to surgery as well, which is excessive at the minute yeah.*’ (P4, man)

Being an elective surgical procedure, orthognathic surgery was often cancelled to give theatre time and space for other critical services. Unfortunately, this led to an increase in surgical waiting time in some units.

The mismatch between supply and demand for orthognathic services was apparent for the excessive waiting time. This mismatch was accounted for two reasons among the sample studied. First, the geographical area covered by the service provider increases the demand compared to supply. Such service providers also faced the challenge of a shortage of service personnel. The second reason was due to a genuine increase in demand among well-educated, dentally fit populations.

‘*Again, it’s really quick for that [clinics] to be cancelled because you can’t backfill it, so we’re desperately trying to have five [MDT clinics] a month for a month, but desperately failing. And then I think the demand has gone up and up the types of patients that we’re seeing, and you know, very high IOTN issues*.’ (P2, woman)

### Theme 5: Use of 3D planning for surgery

Four orthodontists reported 3D planning software was used by the surgeons to plan orthognathic surgery, including Materialise, Synthesis and KLS Martin. The use of 3D planning in most units has been a new development, but those who have used it for long enough have reported feeling as though they had better surgical outcomes. However, others have said they are unsure since it is still early days.

‘*I haven’t, since its very early days and I haven’t seen the outcome yet. Our surgeons love it [3D planning]. They think it’s great and, as I said, we find it easier. However, it is expensive*.’ (P9, man)

Nevertheless, participants who used it and five who aspired to use it all agreed it came with additional financial implications.

‘*We have been trying to go 3D for a while now [. . .] So actually taking cone beam and planning the surgery so on our planning clinic to be able to three-dimensionally see before the patient comes in what movements because I think it would be helpful for the more complex asymmetry and cant cases [. . .] but it’s the financial restraints within the NHS; we’re taking baby steps so that we would like to improve.*’ (P6, woman)

### Theme 6: Need for a psychologist as part of the orthognathic MDT

The presence of a psychologist in the orthognathic MDT was felt to be critical for all the participants. While most participants expressed interest in having a clinical psychologist who is familiar with the management of patients who have facial appearance concerns as a permanent attendee to all MDT clinics, others were happy to have such a named psychologist available as part of the care pathway for the orthognathic patients.

‘*The psychologists have joined our clinic; as they do their job, they are very good at sort of unpacking and almost translating the words that the clinical team are using and the words a patient is using. Really get to the nuts and bolts of what the patient wants and what the team can offer*.’ (P9, man)

Participants who had access to a psychologist were extremely satisfied with the support that was available through their services and stated that all patients seen on orthognathic clinics benefited from the psychologist’s presence.

‘*My experience of it is that you don’t necessarily have someone a psychologist who has much experience in that particular field and then they don’t necessarily pick up on the intricacies or the nuances of what we’re. . . you know. . . we’re talking a lot about it, aren’t we? But we’re not really qualified to deal with it, so we might be thinking this this is ringing alarm bells. But then the psychology team don’t. Not that they don’t agree, but it doesn’t meet their criteria in the same way, so I struggled with that.*’ (P2, woman)

In the absence of a clinical psychologist within the MDT, participants reported using an ad hoc referral service either to CAMHS or to the patient’s general practitioner for support. However, the outcome of such referrals was not always felt to be valuable in supporting their orthognathic care. This was often due to the psychologist’s lack of experience dealing with psychological issues associated with facial appearance.

The majority of participants mentioned attempts to make a business case for having a clinical psychologist on the team but failed due to lack of funding or difficulty in recruitment.

### Theme 7: Non-compliance with British Orthodontic Society (BOS)/British Association of Oral & Maxillofacial Surgeons (BAOMS) minimum dataset for record collection

None of the participants reported 100% compliance with the BOS/BAOMS minimum dataset for record collection. However, some participants had printed copies of the minimum dataset template within the patient’s medical records to check compliance with the record collection.

‘*I am not entirely sure that this guideline is this good. . . that, and I’m thinking that we need to use our resources appropriately and maybe not taking the records which we are not using it, which I’m not going to be.*’ (P18, woman)

‘*I don’t think taking lateral ceph post-debond is in the patient’s best interest*.’ (P7, woman)

It was suggested that the minimum dataset could perhaps be revised to allow better compliance with the record collection.

## Discussion

General themes were identified, which formed a basis in determining the strengths and weaknesses of an orthognathic MDT structure. Overall, this study was able to identify the key areas that were considered to be determinants in a successful orthognathic MDT structure from the clinician’s perspective. This is a novel addition to the literature.

Although the study initially set out to identify characteristics of various orthognathic MDT structures that were clear strengths or weaknesses, it was soon evident that some of the characteristics were considered a strength for some units while being a weakness for others based on how that characteristic eventuated for them. For example, consultants expressed satisfaction about the teaching and training opportunity the orthognathic MDTs provided for orthodontic trainees when it was possible to deliver it. However, in situations where the size of the surgery or bay where the orthognathic clinics were run were of a smaller dimension (especially during the pandemic), accommodating trainees on the clinic for training was seen as a disadvantage. Hence, the themes identified in this study highlight the characteristics of orthognathic MDT setups that are essential for optimising the clinic’s successful running.

MDT clinics’ educational value for specialist trainees has been identified in general and non-technical skills for UK trainees, with trainees learning from participating in case preparation, presentation and discussions (Trivedi, 2019). Attendance at hypodontia MDT clinics has been linked to successful outcomes of the ISFE examination, suggesting that these clinics may be beneficial for developing clinical reasoning (Ali et al., 2016). Similarly, the present study confirms the educational value of orthognathic MDT clinics for trainees as perceived by consultant orthodontists. However, future studies exploring the trainees’ perspective is essential to enumerate the true value of attending orthognathic MDTs.

A sufficiently spacious closed surgery was the preferred location for conducting the orthognathic MDT clinics. The closed surgery was preferred for privacy, and the space available was found to be a vital issue for social distancing and for allowing access to trainees in the clinic, as previously discussed. Considering this study explored the clinician’s perspective of orthognathic MDT design, this finding was unsurprisingly a novel addition to knowledge.

The national orthognathic audit 2017–2018 reported a broad range of waiting time (range <1 month to 4 years) from immediate preoperative MDT to surgery (Ireland et al., 2019). Consistent with this, a longer waiting list/waiting time was a weakness of some of the orthognathic MDT clinics in this study. Clinicians stated the disparity in supply versus demand for orthognathic treatment and short notice cancellation of elective orthognathic surgery as the two reasons for an increase in the waiting list.

The use of 3D planning as part of orthognathic clinics was a theme that was not anticipated, but it was evident that the known reliability and reproducibility of 3D planning (Alkhayer et al., 2020) has influenced the practice of many clinicians in England. However, the additional cost versus the benefits of using 3D planning was seen with scepticism by clinicians who had not used or used 3D planning long enough to comment on it. The clinicians perceived role of 3D planning in orthognathic clinics was a novel and interesting finding of this study.

This study confirmed the findings from previous studies about the benefits of having a clinical psychologist to improve the quality of patient care (Selvaraj et al., 2019), and not all units in England have access to a psychologist as part of their orthognathic MDT (Paul, 2017). Further, the clinicians perceived the lack of a psychologist in orthognathic MDT as a weakness of their MDT design. A recent article described establishing the need for and setting up psychological access in existing orthognathic services (Casey et al., 2021). On the other hand, the close working relationship between the orthodontist and the surgeon who are part of the orthognathic MDT was emphasised in past studies (Cunningham and Johal, 2015; Khechoyan, 2013). The findings of the current study confirmed this as an important characteristic of a successful orthognathic MDT clinic.

The collection of patient records was discussed as part of the orthognathic care pathway (Cunningham and Johal, 2015; Royal College of Surgeons of England, 2013). Further, some participants discussed record collection as part of the orthognathic MDT. Therefore, the record collection and its role in the MDT clinics were explored in this study. It was found that there was a general non-compliance with the minimum dataset record collection as set by BOS/BOAMS. Since developing the minimum dataset for orthognathic records in 2006, its compliance has been unproved (Morris, 2015), but various isolated audits have reported non-compliance with minimum dataset record collection (Dewi et al., 2013; Kwon et al., 2018). In addition, clinicians justified non-compliance based on the need to revise the minimum dataset for orthognathic records collection stating reasons such as lack of justification for postoperative lateral ceph radiographs, burden of five-year postoperative follow-ups and patients’ failure to attending review appointments.

The findings of this study must be interpreted with caution due to its methodological limitation. While the orthognathic MDT consists of other members and orthodontists, only the orthodontists were included in this study. Therefore, the results may not accurately represent the perceptions of all the clinicians in the orthognathic MDT. Further, the researcher did not observe or perceive the correct setting of each orthognathic MDT since data were only obtained via online interviews. Therefore, the actual setting of the clinics has not been accounted for in this study. This limitation can be overcome in a subsequent ethnographic study.

## Conclusion

In conclusion, the characteristics of an orthognathic MDT that influence its successful function have been highlighted. Like many recent studies, orthodontic consultants in this study have recommended the presence of a psychologist in the team for a successful orthognathic MDT. Size and privacy available in the clinical setup influenced teaching and training of the specialist trainees and orthognathic patient care. A close working relationship of the team and shorter waiting list were positive characteristics of the orthognathic MDT. Non-compliance with the orthognathic minimum dataset record collection was seen, and a revision of the minimum dataset was recommended. Finally, a general interest in using 3D planning for orthognathic treatment was found in keeping with technological advancement.

## Supplemental Material

sj-pdf-1-joo-10.1177_14653125231165069 – Supplemental material for Characteristics of orthognathic multidisciplinary team clinics in England. Part 2: A qualitative study Click here for additional data file. Supplemental material, sj-pdf-1-joo-10.1177_14653125231165069 for Characteristics of orthognathic multidisciplinary team clinics in England. Part 2: A qualitative study by Ninu R Paul and Sarah L Rolland in Journal of Orthodontics
